# The evolutionary function of conscious information processing is revealed by its task-dependency in the olfactory system

**DOI:** 10.3389/fpsyg.2014.00062

**Published:** 2014-02-05

**Authors:** Andreas Keller

**Affiliations:** Philosophy Program, Graduate Center, The City University of New YorkNew York, NY, USA

**Keywords:** olfaction, consciousness, evolution, task-dependency, information processing

## Abstract

Although many responses to odorous stimuli are mediated without olfactory information being consciously processed, some olfactory behaviors require conscious information processing. I will here contrast situations in which olfactory information is processed consciously to situations in which it is processed non-consciously. This contrastive analysis reveals that conscious information processing is required when an organism is faced with tasks in which there are many behavioral options available. I therefore propose that it is the evolutionary function of conscious information processing to guide behaviors in situations in which the organism has to choose between many possible responses.

Brains continuously process information. Sometimes this information processing is conscious, which means that there is *something it is like for the organism to process the information*, to borrow an expression from the philosopher Thomas Nagel (1974). In contrast, most information is processed non-consciously and there is nothing it is like for the organism to process the information, just like there is nothing it is like for the organism to filter blood in the kidney. In this paper I will contrast conscious and non-conscious processes in the olfactory system to identify the evolutionary function of conscious information processing.

The approach presented here differs in two important aspects from similar approaches. First, this is not an analysis of the *function of consciousness* but of the *function of conscious information processing*. Conscious information processing is the subset of information processing that is accompanied by consciousness. An analogy can illustrate the importance of this seemingly subtle difference. The central nervous system can be divided into gray matter and white matter. One can speculate about the function of the “grayness” of gray matter, or one can investigate the function of gray matter without discussing the function of its “grayness.” The approach presented here is analogous to the second option. This means that whatever conclusion will be reached, it is consistent with consciousness having no function at all (Flanagan, 1997). A second important feature of the approach presented here is that I am interested in the *evolutionary function *of conscious information processing, whereas discussions of the function of consciousness often focus on the *current uses *of consciousness. To illustrate the importance of the distinction between evolutionary function and current uses, let’s consider wings. The evolutionary function of wings is flight. However, wings have many different current uses. Bees use wings to communicate. Some birds protect their young by taking them under their wings. Butterflies have patterns on their wings to scare or confuse predators. Male ostriches and birds of paradise use their wings during courtship displays and cranes use their wings to shade the water surface to better see their prey swimming underneath (Gazzaniga et al., 2009, p. 651).

I believe that the analysis of the evolutionary function of conscious information processing presented in this paper will provide an interesting contrast to the literature on the current uses of consciousness. My analysis will be presented in biological terms because determining evolutionary functions of evolved mechanisms is a common strategy in biology where “mechanism” and “evolution” are central concepts. I will introduce the strategy of determining evolutionary function through contrastive analysis in the first section of the paper. In the second section I will apply this strategy to conscious information processing in the olfactory system. The result of this analysis is that it is the function of conscious information processing in the olfactory system to guide behaviors in situations in which an organism is faced with tasks in which there are many different behavioral options to chose from. In the third section I will argue that many apparently competing proposals describe the processes in the brain at a different level and are therefore consistent with the results presented here. In the fourth section I will sketch how the task-dependency of conscious information processing relates to more general observations outside of the olfactory system.

## CONTRASTIVE ANALYSIS OF EVOLUTIONARY FUNCTION

The evolutionary function of a biological mechanism is determined by its evolutionary history. At some point during our evolutionary history, an organism appeared that had the capacity to process information consciously in a certain brain structure. This capacity was inheritable, which means that the organism’s offspring also had the capacity. In addition, the organism with the capacity for conscious information processing had an adaptive advantage over organisms without this capacity. Conscious information processors therefore had more offspring (on average over time) than other organisms of the same species. If a capacity is heritable and results in an increased number of offspring, it will be selected for through evolution by natural selection (Lewontin, 1985). To identify the evolutionary function of conscious information processing, one has to identify the reason why the first conscious information processor had more offspring than its competitors.

From this characterization of evolutionary function it is clear that the evolutionary function of conscious information processing is not necessarily something that could not also be accomplished by non-conscious processes. As has been pointed out previously (Dretske, 1997), the lack of alternatives is not a requirement for evolutionary function. The function of a fish’s fins is aquatic locomotion although many mammals, birds, insects, amphibians, jellyfish, and other creatures move in water without fins. This is important to point out because inquiries into the function of consciousness are often attempts to identify functions that can only be performed consciously. However, the lack of alternatives is not part of the concept of evolutionary function. It is also not reflected in the common usage of the word “function.” That one can sit on rocks, benches, and toilets does not conflict with the proposal that it is the function of chairs to provide a surface to sit on. Similarly, that the first organism capable of conscious information processing had an advantage over organisms without that capability does not mean that this organism was capable of solving problems its competitors could not solve. The more likely scenario is that conscious information processing was more efficient than non-conscious information processing at solving certain problems. Evolutionary processes optimize efficiency. Efficient information processing is achieved by keeping the brain as small as possible. The metabolic rate in brain tissue is much higher than in other tissues, because the membrane potential of neurons has to be permanently sustained (Nieuwenhuys et al., 1998, p.11–14). In humans, around 20% of all energy is consumed by the brain, a ten times higher percentage than in other mammals like pigs and horses (Mink et al., 1981). Therefore, even if the problems that are solved in our brains by conscious information processing can be solved in a larger brain with non-conscious information processing, there is strong adaptive pressure to process information consciously.

The interesting question is, what the problems are that are more efficiently solved using conscious information processing. The fact that we still process much information non-consciously despite having the capacity to process information consciously suggests that conscious information processing is not simply a generally superior way of processing information. Instead, it is likely that different strategies are most efficient for different tasks. It is not uncommon that there is more than one information processing strategy for solving a problem. Often a strategy for exact calculations and an alternative strategy for estimations are available. In statistics there are for example two options to compute the *p*-value of a contingency table. One option is the Fisher’s exact test and the other is the Chi-square test. As the name suggests, the Fisher’s exact test results in the exact *p*-value. The Chi-square test, in contrast, is an approximation. The larger the sample size, the closer to the exact value is the Chi-square test approximation. In addition the number of calculations required to arrive at the exact value increases with the sample size. If one assigns a cost for calculations and a cost for potential inexactness of the *p*-value, then one can calculate the less costly strategy for determining the *p*-value for any sample size. For small sample sizes the Fisher’s exact test is more efficient than the Chi-square test. However, because with increasing sample size the difference in calculation cost increases and the approximation approaches the exact value, there is a sample size above which the Chi-square test is more efficient. At very large sample sizes, the calculation cost of the Fisher’s exact test is prohibitive.

Now let’s imagine an evolved biological system whose fitness depends on calculating *p*-values of contingency tables. If there are variants that process the information for small sample sizes using a Fisher’s exact test and for large sample sizes using a Chi-square test, these variants will have an evolutionary advantage over variants that always use the Chi-square test as well as over variants that always use the Fisher’s exact test. The variants that can switch between the two computations cannot do anything that their competitors cannot do, but they do these things more efficiently. The evolutionary function of the Chi-square test performing mechanism would in this scenario be to calculate *p*-values for contingency tables when the sample size is large.

The methodology to identify the evolutionary function of a biological mechanism is contrastive analysis. Contrastive analysis compares situations in which the mechanism under study is employed to situations in which alternative mechanisms are employed. For fish’s fins, this methodology would result in identifying aquatic locomotion as the fins’ evolutionary function. Contrastive analysis requires generalizations over many cases and because evolution is an ongoing process the correlation between traits and functions is not expected to be perfect. The fact that some animals without fins are capable of aquatic locomotion does not mean that aquatic locomotion is not the function of fins. Furthermore, aquatic locomotion is the evolutionary function of fins even though a contrastive analysis is likely to uncover that sometimes fins are not used for aquatic locomotion but for walking over land, courtship displays, or temperature regulation. It is vey common for structures or mechanisms that evolved for one function to be further adapted for additional functions. The goal of contrastive analysis is to analyze current uses to identify the phylogenetically earliest function of a structure or mechanism. If the evidence shows that the first animals with fins used them for aquatic locomotion, then aquatic locomotion is the evolutionary function of fins.

To identify the evolutionary function of conscious information processing, situations in which information is processed consciously have to be compared to situations in which information is processed non-consciously. Numerous experimental designs have been developed for this purpose (Kim and Blake, 2005). One common strategy is to use two similar stimuli, only one of which is processed consciously. In vision, short visual displays are often contrasted with longer displays. Another often used paradigm is visual masking, in which a two-part stimulus that consists of a “target” and a “mask” is differently processed from a stimulus that consists only of the “target” (Breitmeyer, 2008). In olfaction, the processing of low concentrations of an odor can be compared to the processing of higher concentrations. Unfortunately, these experiments are very difficult to interpret because it is challenging to, for example, distinguish between the hypothesis that an odor at low concentration is not consciously processed *and therefore* cannot be named and the alternative hypothesis that the odor is not consciously processed *and also* cannot be named. For this reason, contrastive analysis of the processing of the same physical stimulus is preferable (Kim and Blake, 2005). The most common experimental design in which the same visual stimulus is processed differently involves visual competition (Blake and Logothetis, 2002). Examples of visual competition are ambiguous figures and binocular rivalry (Tong et al., 2006). Another situation in which the same visual stimulus is processed in different ways is when spatial attention is shifted (Cave and Bichot, 1999; Chun et al., 2011). Of special interest are covert shifts of visuospatial attention (as opposed to overt shifts which depend on eye movements; Wojciulik et al., 1998). These experimental designs are superior to those in which the processing of two different stimuli is compared, however, they do not contrast non-conscious with conscious information processing. Instead, they contrast conscious processing of one content with conscious processing of another content. For example the duck-rabbit, an ambiguous figure that can be perceived as a duck or as a rabbit, can be used to compare the conscious processing of the image of a duck with the conscious processing of the image of a rabbit.

The ideal situation for contrastive analysis is when both the stimulus and the content are the same, but processing is conscious in some situations and non-conscious in other situations. The sound of a clock ticking is sometimes processed consciously and sometimes processed non-consciously. One can become suddenly aware of it (Block, 1997). The same is true for the feel of one’s clothes touching the skin. These are cases in which the same stimulus and the same content is sometimes processed consciously and sometimes non-consciously.

## CONSCIOUSNESS IN THE OLFACTORY SYSTEM

The olfactory system is well suited for identifying the evolutionary function of conscious information processing because it is common that the same olfactory information is processed consciously in some situations and non-consciously in other situations. The olfactory system has the further advantage that it is anatomically and computationally relatively simple (Haberly, 2001; Lledo et al., 2005; Sela and Sobel, 2010). It has even been suggested that it represents the minimal neuroanatomy that is required for conscious information processing (Morsella et al., 2010). There is only a single synapse between the odor stimulus and the olfactory cortex and the pre-cortical processing in the olfactory bulb is understood in great detail (Shepherd et al., 2004; Hintiryan et al., 2012). Furthermore, the human olfactory system is an evolutionary conserved structure (Eisthen, 1997) that presumably has not changed significantly since it first evolved the capacity to process information consciously.

Despite the relative simplicity of information processing in the olfactory system and the evolutionary ancient neuroarchitecture underlying it, sometimes information about our olfactory environment is processed consciously. However, reflecting the relative simplicity of our olfactory system, olfactory phenomenology is very simple and lacks many of the complexities that hinder our understanding of conscious perception in the visual modality (Lycan, 2000; Köster, 2002; Stevenson, 2009a). Most prominently, the spatial structure of olfactory phenomenology is very impoverished. Subjects cannot discriminate between a stimulus in the left and right nostril (Radil and Wysocki, 1998; Frasnelli et al., 2008) and, despite the fact that olfactory experience can have a diachronic spatial structure, many philosophers think that olfactory perception does not represent the location or direction of olfactory stimuli (Lycan, 2000; Smith, 2002; Matthen, 2007; Peacocke, 2008; Batty, 2010). The phenomenological and biological simplicity of the human olfactory system and the fact that it is phylogenetically old and evolutionary conserved make it a good system for identifying the evolutionary function of conscious information processing by comparing situations in which olfactory information is processed consciously to situations in which it is not, which is the goal of this section of the paper.

Humans mostly use their sense of smell to evaluate food, ambient air, and potential mates (Stevenson, 2009b). Often, olfactory evaluation does not require conscious information processing. This is reflected by a variety of olfactory metaphors for situations in which we evaluate something but are not conscious of our reasons for the outcome of the evaluation: “smell a rat,” “something smells fishy,” “smell test,” etc. That olfactory evaluation does not always require information to be processed consciously has also been demonstrated empirically. Social preferences, for example, have been shown to be influenced by odors that were not consciously processed by the subjects (Li et al., 2007). Similarly, an odor-specific effect has been shown on judgments of participants posing as job candidates (Cowley et al., 1977). Like evaluation of other people, evaluation of food often does not require conscious information processing. For example, sucrose solution is evaluated to be sweeter when an undetectably small amount of ethyl butyrate is added (Labbe et al., 2006). Similarly, odors at concentrations that are too low to be consciously processed can change the perceived odor quality when added to a mixture (Guadagni et al., 1963; Ito and Kubota, 2005). In all these cases, conscious information processing is not required for evaluation. However, there are also tasks in which conscious information processing is required. If the decision that has to be made is to either swallow or spit out a sip of wine, conscious processing is not required. However, if the task is to write a review of the wine’s flavor, it is necessary to process the sensory information consciously.

Humans use their sense of smell predominantly for evaluation, however, this is a recent evolutionary development. In many other vertebrates odor-dependent navigation is the most prominent odor-guided behavior (Jacobs, 2012). In humans, odor-guided navigation does not play an important role, but there are still some examples of it. Infants, for example, use olfactory cues to orient toward their mother’s breast (Varendi et al., 1994; Varendi and Porter, 2001). Under experimental condition, humans are also surprisingly good at following an odor trail (Porter et al., 2006). Navigating physical space based on olfactory cues does usually require conscious processing of the olfactory information. The only strategy available to locate the source of the gas leak in a building is through serial sampling and comparisons [Unlike other species, humans to not have the capacity for directional smelling by comparing the olfactory input of the two nostrils (Radil and Wysocki, 1998; Frasnelli et al., 2008; Kleemann et al., 2009)]. To locate the gas leak, one has to sample the air by sniffing while walking from room to room. Through intensity comparisons, the location of the gas leak can be identified (Richardson, 2011). Throughout the entire process olfactory information is processed consciously and compared to stored conscious percepts of the smell in the other rooms. It seems unlikely that this task could be accomplished without conscious information processing. On the other hand, there is evidence that odor-dependent place preferences can be mediated without conscious information processing. It has been shown that people chose chairs in a dentist’s waiting room depending on the odor the chairs were perfumed with (Kirk-Smith and Booth, 1980; Pause, 2004). In other studies, releasing an odor among the slot machines on the casino-floor of the Las Vegas Hilton increased how much was gambled in that area (presumably by increasing the time gamblers spent in the area; Hirsch, 1994). Perfuming a small pizzeria in the Brittany region of France with lavender increased the time patrons spend in the restaurant as well as the amount of money they spent (Guéguen, 2006). Many studies of the effect of ambient scents on behaviors do not control for all potential biases (Teller and Dennis, 2012) and subject numbers are usually low and replications rare. Each individual study has therefore to be interpreted with care. However, I think that taken together there is good evidence that we prefer to spend time in a pleasantly scented area than in an unpleasantly scented area, and that this preference can be mediated through non-conscious processing.

This brief overview over olfactory behaviors shows that olfactory information processing has two main functions in humans: navigation and evaluation. Contrastive analysis shows that for both functions there are situations in which they can be accomplished without conscious information processing and situations in which conscious information processing is required. We will swallow good wine and spit out wine that has turned into vinegar without the need to process the sensory information consciously. On the other hand, the very same sensory information has to be processed consciously when it is our task to write a review about the wine. Similarly, we will pick the seat in a room furthest away from an unpleasant smelling individual without the need for conscious information processing, but locating a gas leak requires conscious processing of the olfactory information. The difference between situations in which conscious processing of olfactory information is required and situations in which it is not required is therefore a difference in the tasks the organism is facing. Whether the sensory information has to be processed consciously or not depends on what the information is to be used for.

The salient difference between the tasks for which conscious information processing is required and the tasks for which it is not required is the number of behavioral options between which the organism has to choose to accomplish the task. In the case of spitting out or swallowing the wine, there are two options: spitting it out or swallowing it. In the case of writing a review about the wine, if the reviewer has a vocabulary of 10,000 words and the review is 100 words long, there are 6.5 × 10^241^ options. Similarly, in the case of having a place preference based on an odor, there are only two options: stay/go. However, in the case of attempting to identify the source of an odor there are as many options as there are paths in two-dimensional space. These examples show that the number of behavioral options increases in a combinatorial manner. There is a limited number of words and the task of writing a review consists of deciding between the many possible combinations of these words. Similarly, every navigation in space is a combination of many stay/go/turn decisions. It is tasks that require these combinations that require conscious information processing.

It may seem easy to find counterexamples that contradict this conclusion. We sometimes process the soothing lavender odor consciously when we are lying on the massage table in a spa and the only behavioral option that we consider is to do nothing. However, these apparent counterexamples are based on a confusion between evolutionary function and current use. That the crane uses its wing to shade the water surface to better see its prey is not in conflict with the proposal that the evolutionary function of wings is flight. Similarly, the current use of conscious information processing in a wide variety of situations does not contradict the proposal presented here. The result of the contrastive analysis is therefore that whether olfactory information is processed consciously or not depends on the task that the organism is facing. The stimulus has to be strong enough (very low concentrations of odors cannot be processed consciously) and the organism has to be in the right state (an organism in a coma cannot process information consciously), but when these requirements are met, whether information is or is not processed consciously depends on the task the organism is facing. In evaluation as well as in navigation, information is processed consciously when the organism is faced with many different behavioral options, but non-consciously when the choice is between few behaviors. Verbal communication and goal-directed navigation in physical space are combinatorial tasks with a very large number of options, which is why they require conscious information processing.

## DESCRIPTIONS OF BRAIN PROCESSES AT DIFFERENT LEVELS OF HIERARCHY

I have developed a proposition about the function of conscious information processing that is consistent with the facts about consciousness in the olfactory system. The same facts are also consistent with a variety of other proposals. In this section of the paper I will discuss two of these alternative proposals and argue that they are not in conflict with the proposal presented here because they describe the processes in the brain at a different level of hierarchy.

Biological systems are hierarchically organized. Atoms make up molecules, which are the building blocks of cells. Cells combine to functional units that are called organs, and organisms are collections of organs. Organisms are parts of populations, which are parts of ecosystems. The collection of all ecosystems is the biosphere. Consequently, different processes can be described at different levels. There are textbooks of cognitive neuroscience and textbooks of molecular neuroscience. The topic of these textbooks is the same, but they address the topic at a different level of description. Statements at different levels of description cannot be in conflict. “Neurotransmitter release in neuron X leads to an increase in calcium level in neuron Y,” “Neurotransmitter release in neuron X leads to firing of neuron Y,” and “Neurotransmitter release in neuron X leads to avoidance behavior.” are not competing hypothesis but descriptions of the same phenomenon at the molecular, cellular, and behavioral level.

For the purpose of this paper, the processes in the brain are described as information processing. This level of description is one level above the cellular or neural circuit level. Information processing in the brain is a consequence of neural activity in neural circuits that evolved for the purpose of processing information. This is the level at which much recent progress in the neurosciences has been made. The level of description above information processing is the level of cognitive processes. This is the level at which much recent progress in consciousness research has been made. There are therefore several proposals about consciousness at the level of cognitive processes, some of which are consistent with the data reviewed in this paper. However, these proposals are not in conflict with the proposal presented here. Instead, they are descriptions at a different level of the biological hierarchy.

One such proposal is that it is the function of *higher cognitive processes* to guide behaviors in situations in which there are many different behavioral options. A striking difference between the task of either drinking a wine or not and writing a wine review is that writing a review requires higher cognitive processes. Semantic symbols (words) have to be combined in ways that conform to the rules of syntax. Examples like this have lead some to believe that syntactic thought plays an important role in consciousness (Rolls, 2007). On the other hand, to locate an odor, physical space has to be represented and a multi-step path through it has to be planned. Examples like this have lead to proposals that phenomenal space is necessary for consciousness (Revonsuo, 2006, p. 170). Visually representing physical space and syntactic thought are very different cognitive processes. However, both can be considered “higher cognitive processes” and then the result of the contrastive analysis, at the level of cognitive processes, is that it is the function of higher cognitive processes to guide behaviors in situations in which there are many different behavioral options.

Another proposal at the level of cognitive processes that follows from the contrastive analysis presented here is that it is the function of *attention* to guide behaviors in situations in which there are many different behavioral options. A high level of attention is engaged when we are asked to report about the flavor of wine and when we try to locate a gas leak. Less attention is required to detect spoiled food one is about to swallow.

Both of these proposals, and maybe also others, are consistent with the results of the contrastive analysis presented here. However, they are descriptions of brain processes at the level of cognitive processes, and they are not in conflict with proposals at the level of information processing like the one presented here. Instead, these proposals raise the interesting question of the relationship between the information processing level and the cognitive system level. I have previously argued for a close connection between attention and conscious information processing in the olfactory system (Keller, 2011). However, this view is not universally shared, and in visual perception cases of consciousness in the absence of attention (van Boxtel et al., 2010) and attention in the absence of consciousness (Norman et al., 2013) have been described. The mapping of descriptions on the information processing level and the cognitive level therefore does not seem to neatly respect the borders of the categories that have been employed at the two different levels of description.

## BEYOND OLFACTORY CONSCIOUSNESS

According to the proposal defended here, conscious information processing has been selected by evolutionary processes because it is more efficient than non-conscious information processing at solving tasks in which there are many behavioral options. It is plausible that in situations with few potential behaviors a simple algorithm that has been shaped by innate preferences and a combination of associative learning and generalizations is an efficient way of approaching the task. Such an algorithm would however not be an efficient way for approaching the task of writing a wine review because for this task it would be required to associate each possible flavor with one of the extremely large number of possible reviews. Instead, it may be that the most efficient way of writing a review, or of navigating toward a goal, is to simulate the responses before executing them. According to this theory, conscious information processing creates a simulation of the world in which behaviors can be tried out without actually being performed (Hesslow, 2002). A metaphor that has been used for this type of information processing has called it a “virtual reality arena of the mind” (Revonsuo, 2006). The metaphor of the “virtual reality arena of the mind” is similar to the influential metaphor of the “theater of the mind” (Baars, 1988, 2005). The key difference between a virtual reality arena and a theater is that the play in the virtual reality arena is interactive. Virtual reality arenas are computer-simulated interactive three-dimensional environments and if this metaphor is taken too literal, there is the danger of interpreting it as showing that phenomenal space is necessary for consciousness (Revonsuo, 2006, p. 170). However, navigation in physical space is only one type of task that has so many behavioral options that it is most efficiently solved by conscious information processing. Immersion in a virtual reality arena can simulate navigation in space, but it cannot simulate writing a wine review. A better metaphor that can be applied to all tasks that require conscious information processing, not only to navigation in physical space, is “menu for action,” which has been suggested by Prinz (2012). Conscious information processing, according to Prinz, is, like the virtual reality arena, a precondition for decision rather than a mechanism for decision.

I have so far discussed only odor-guided behavior to provide support for the proposal that it is more efficient to process information consciously (and thereby create a menu for action), when there are many options. In situation with few options, I propose, it is more efficient to process information non-consciously. I have argued that odor-guided behaviors are a good model system for consciousness research. However, only a very small portion of behaviors are odor-guided. In the last section of this paper I will briefly discuss task-dependency of conscious processing of non-olfactory information. A comprehensive survey of non-olfactory information processing is beyond the scope of this paper, but the discussion of the non-olfactory cases will help clarify the proposal presented here.

The proposal that information is processed consciously when an organism is faced with many behavioral options explains why there are large differences in the frequency with which information is processed consciously between different modalities. There are almost always enough odor molecules in the air that we inhale to activate our olfactory system. However, we only rarely process this information consciously. In contrast, we usually process at least some visual information consciously. The reason for this difference between the modalities is that the behaviors that are visually guided are usually more complex than those that are odor-guided. Vision is the dominant sense in humans because it represents physical space more accurately than the other senses. Behaviors that depend on precise movements in physical space, like manipulation of objects and tool use, usually require choosing between a large number of behaviors and the visual information that guides these behaviors is therefore most efficiently processed consciously. In contrast, as pointed out above, olfaction mostly guides evaluative behaviors, which are usually associated with binary decisions like stay/go, spit/swallow, inhale/hold your breath, or approach/avoid.

Another feature of conscious information processing that is consistent with the dependency of conscious information processing on the number of behavioral options is that during skill acquisition information has to be processed consciously whereas during skill retrieval the same information can efficiently be processed non-consciously (Schneider et al., 1994; Floyer-Lea and Matthews, 2004). As someone learns to play a new song on the guitar, they have to process their finger positions and movements consciously. However, as they become more familiar with the song, the finger movements can increasingly be guided by non-conscious information processing. The reason for this change in how the information is processed is that familiarization with the song decreases the number of behavioral options. When a song is played from sheets for the first time, at every point during the song there is a very large number of combinations of notes and therefore finger movements that may follow. Once the song is familiar, at every point during the song, there is only one salient sequence of finger movements that follows. The potential behaviors are reduced from a very large number of combinations to one, which results in a difference in how the information is processed. The same reduction in the number of behavioral options is accountable for the difference between driving a route for the first time and driving it for the hundredth time. When driving a very familiar route in low traffic, not much sensory information is processed consciously. However, if suddenly a deer jumps onto the road in front of the car, information has to be processed consciously, because the deer makes it necessary to consider a wide variety of possible responses to avoid a collision. These examples illustrate that the relevant number is not the number of all possible behavioral options, but the number of task-relevant options. When pouring liquid in one’s mouth with the goal of reducing thirst, the task-relevant options are to swallow it or to spit it out. Writing a review about the taste of the liquid is also an option, but it is not task-relevant. When a deer jumps in front of one’s car, the number of task-relevant options suddenly increases and information is therefore processed consciously.

## CONCLUSION

A contrastive analysis of situations in which olfactory information is processed consciously and situations in which it is processed non-consciously suggests that information is processed consciously only when an organism is faced with a task that requires considering many different behaviors. This appears to be the evolutionary function of conscious processing of olfactory information. Although task-dependency of conscious information processing is also widespread outside of the olfactory system, the proposal presented here has to be tested extensively outside of olfaction to see if it generalizes to conscious information processing in general. Regardless of the outcome of these tests, I hope that the analysis of the *evolutionary function of conscious information processing* presented in this paper will provide an interesting addition to the literature that is dominated by analyses of *current uses of consciousness*. Neuroscience has made remarkable progress in understanding brain processes at the level of neuronal circuits. Consciousness research has investigated consciousness mainly at the level of cognitive processes. An analysis at the level of information processing that lies between these two levels of hierarchy will hopefully help to bring these two fields together.

## Conflict of Interest Statement

The author declares that the research was conducted in the absence of any commercial or financial relationships that could be construed as a potential conflict of interest. The review editor D. Rosenthal declares that, despite being affiliated at the same institution as the author Andreas Keller, the review process was handled objectively and no conflict of interest exists.
